# Memantine augmentation of sertraline in the treatment of symptoms and executive function among patients with obsessive-compulsive disorder: A double-blind placebo-controlled, randomized clinical trial

**DOI:** 10.1186/s12888-021-03642-z

**Published:** 2022-01-12

**Authors:** Sanaz Askari, Saba Mokhtari, Seyed Vahid Shariat, Behnam Shariati, Masoomeh Yarahmadi, Mohammadreza Shalbafan

**Affiliations:** 1 Mental Health Research Center, Psychosocial Health Research Institute (PHRI), Department of Psychiatry, School of Medicine, Iran University of Medical Sciences, Tehran, Iran; 2 Department of Psychiatry, University of Social Welfare and Rehabilitation, Tehran, Iran; 3 Brain and Cognition Clinic, Institute for Cognitive Sciences Studies, Tehran, Iran

**Keywords:** Glutamate, Memantine, Obsessive-Compulsive Disorder, Randomized controlled trial

## Abstract

**Background:**

Medications currently recommended for the treatment of Obsessive-Compulsive Disorder (OCD) usually decrease the severity of the symptoms by 20–30%; however, 40–60% of OCD patients do not achieve a satisfactory response. Our main objective was to investigate the effectiveness of memantine, a non-competitive N-Methyl-D-aspartate (NMDA) receptor antagonist, as an adjunct therapy to sertraline, a selective serotonin reuptake inhibitor (SSRI), to improve severity of symptoms and executive function among patients with obsessive-compulsive disorder.

**Methods:**

Seventy patients with OCD according to the Diagnostic and Statistical Manual of Mental Disorders (DSM–5) criteria, and a Yale-Brown obsessive compulsive scale (Y-BOCS) score of more than 21 were recruited to the study. They received sertraline (100 mg daily initially followed by 200 mg daily after week 4) and either memantine (10 mg twice daily) or placebo in a placebo controlled, double-blinded, parallel-group, clinical trial of 12 weeks. The primary outcome was OCD symptoms measured by the Y-BOCS. Moreover, executive function of participants was measured by the Wisconsin Card Sorting Test (WCST).

**Results:**

The total score, and obsession and compulsion subscales of Y-BOCS significantly dropped in both groups with no significant difference between the two groups. However, memantine group showed a greater response in the number of completed categories subscale of the WCST (p value<0.001). We did not observe any major adverse effects in any of the groups.

**Conclusion:**

Memantine has an acceptable safety and tolerability in patients with OCD and might have a positive effect on their executive function. Nevertheless, the current results don`t support the efficacy of memantine as an adjunctive agent to sertraline for symptoms in patients with OCD.

**Trial registration:**

The trial was registered at the Iranian Registry of Clinical Trials on 04/10/2019 (www.irct.ir; IRCT ID: IRCT20170123032145N4).

## Background

Obsessive-Compulsive Disorder (OCD) affects 1–3% of worldwide population [1, 2]. OCD is characterized by recurrent, unwanted and intrusive thoughts, urges or images which causes anxiety and discomfort, and/or by repetitive behaviors or mental acts that tries to prevent or reduce the associated anxiety [3, 4]. If left untreated, the course is generally chronic [5]. OCD severely impairs quality of life and causes impairment in all aspects of daily functioning [6, 7]. It has been shown that patients with OCD have significant differences with unaffected individuals, in tests related to verbal memory, psychomotor speed, global attention, and visuospatial and executive functions, indicating poorer performance [8, 9]. While patients with OCD have different impairments in multiple aspects of cognition, former studies have shown that one of the most frequently proposed neuropsychological deficits is set shifting [10–14] and one of most important cognitive impairments in OCD is executive dysfunction [8, 12, 15–17]. Furthermore, a positive correlation has been shown between executive function and insight in patients with OCD [18]. Considering the importance of insight in onset, duration, severity, comorbidity, treatment adherence, and overall prognosis of OCD [19–21], this connection could be an important subject that needs further studies. Cognitive impairments, particularly deficiencies in executive functions and information processing, considerably suppress patient’s abilities to gain, maintain and relearn the skills needed for suitable performance of everyday tasks and real-life functioning in OCD patients [10–12, 17].

Currently, selective serotonin reuptake inhibitors (SSRIs) and/or cognitive behavioral therapy (CBT), are considered to be first-line treatments for OCD [22, 23]. Notwithstanding the effectiveness of CBT as a non-pharmacological treatment, it has several disadvantages such as delayed clinical response, limited access, and high cost [24]. SSRIs usually reduce the Obsessive-Compulsive Disorder symptoms by as much as 20–30% and in only 40–60% of the patients with OCD satisfactory treatment is obtained [25, 26].

The cortico-striato-thalamo-cortical (CSTC) circuits, driven by the excitatory neurotransmitter glutamate, are described to be involved in OCD [27]. Glutamate has an important role in many physiological processes including memory, cognition and learning [28]. Striatum is an important brain region in the pathophysiology of OCD and is responsible for motor and cognitive actions [29]. Glutamatergic neurons are the most abundant neurons in cell migration within striatum and migration is also tightly controlled by glutamate [30]. Some studies have shown that glutamatergic over-activity, increased glutamate levels in cerebrospinal fluid (CSF), and polymorphism of N-methyl-D-aspartate (NMDA) receptor’s gene coding, play a part in OCD occurrence [31–34]. Due to high proportion of resistance to SSRI treatment, the focus has shifted to the effect of glutamate and the CSTC brain circuit [35]. Furthermore, there is evidence suggesting that the temporal lobe (TL) has an important role in the pathogenesis of OCD [36–38].

Studies on some glutamatergic drugs have shown different results. In only one RCT on Glycine in OCD patients, it was very intolerable and there was a high rate of drop-outs [39]. Older studies on N-acetylcysteine (NAC) showed some promising effect [40] but more recent studies did not confirm the previous findings and didn’t show an acceptable efficacy [41, 42]. Randomized controlled trials on Riluzole did not show a positive result either [43, 44]. Moreover, studies on D-Cycloserine (DCS) were not successful and did not show a promising efficacy [45, 46]. One study assessed L-carnosine’s effect on patients with OCD as adjunct therapy to fluvoxamine and found significant effectiveness [25]. First two studies on topiramate have shown some positive effects (one on all of the symptoms and the other only on compulsions) but a more recent study did not show a benefit [47–49].

Memantine is a non-competitive NMDA receptor antagonist approved for Alzheimer's disease in many countries, with a good safety profile, and it has also been studied in a variety of psychiatric disorders [50]. It may decrease hyperactivity of the direct pathway of CSTC [35]. Memantine has a targeted de-excitation effect in the temporal lobes on the glutamatergic system and connected brain regions, that might further reduce OCD symptoms [37]. The specific effect of memantine on temporal lobe can be even more helpful in certain patients and subtypes with specific deficits in cognition and maladaptive compensatory memory processes [35].

Open-label studies [51], single [52] and double-blind randomized controlled trials (RCTs) [53, 54] and one systematic review and meta-analysis [55] showed benefits of adding memantine to ongoing SSRI in the treatment of OCD. These RCTs are valuable; however, duration of one of them was not long enough to appropriately assess the OCD symptoms [56] and the other one had a small sample size, a major gender difference in the groups and did not augment memantine to a single drug (there were different SSRIs and clomipramine in the study) [57].

Considering the methodological issues of the abovementioned trials and the resulting inconsistent findings, as well as a lack of studies on the effect of memantine on cognitive impairments in OCD patients [29], we aimed to investigate the benefits of augmenting sertraline with memantine or placebo in reduction of OCD symptoms and cognitive impairments in OCD patients .

## Methods

### Trial setting and design

A 12-week, randomized, double-blind, placebo-controlled, parallel-group trial was performed at the outpatient clinics of Iran Psychiatric Hospital and Tehran Institute of Psychiatry (affiliated with Iran University of Medical Sciences, Tehran, Iran) from January to December 2020.

Participants were randomized to groups with a random permuted block method (ratio of 1:1 and blocks of four). The allocated group of each participant was printed sequentially and enveloped in a non-transparent and sealed envelope similar in appearance, using the random permuted block. The allocation was not in reach of the participants and outcome assessors. The outcome assessor, randomizer, and statistical analyzer each were separate individuals and all of them were blinded to allocation. Additionally, memantine and placebo tablets were similar in size, shape, color, and odor.

### Participants

Patients, aged 18–60 years, with a clinical diagnosis of OCD based on the Diagnostic and Statistical Manual of Mental Disorders, 5th Edition (DSM-5) criteria, were screened for the study [35]. Those with a Yale–Brown Obsessive Compulsive Scale (Y-BOCS) score of ≥ 21 (moderate to-severe OCD) were included [36].

The patients attending to the clinics were consecutively checked for the inclusion criteria and recruited until the sample size was achieved. All of the patients enrolled in the study were assessed with a structured clinical interview designed in accordance with the DSM-5 by an expert psychiatrist [35].

The exclusion criteria were :1) comorbid axis I disorders; 2) a life threatening psychiatric symptoms (such as suicidal ideation); 3) serious medical or neurological conditions; 4) mental retardation (based on clinical judgment); 5) substance dependence (other than nicotine) 6) pregnancy/breast feeding; 7) history of severe allergy to or contraindication for the use of memantine or sertraline; 8) history of complete response with sertraline 9) history of previous psychosurgery for OCD; 10) history of treatment-refractory OCD. During the conduction of the trial, patients were not permitted to participate in any psychotherapeutic treatment. Furthermore, patients were excluded if they used any psychotropic drugs in the last 6 weeks.

### Interventions

Eligible participants were randomized to receive either memantine, 10mg twice per day (start with 5mg daily and increase slowly), or placebo for 12 weeks. All participants, regardless of group assignment, 100 mg/day for 4 weeks (start with 25mg) and then gradually increased to 200mg/day. To minimize the side effects, the dosage of sertraline was slowly increased every week.

### Outcome

Y-BOCS was used for assessment of patients at baseline and at weeks 0, 4, 8, and 12 of therapy. Y-BOCS provides a rating scale for severity of obsessive-compulsive symptoms [54, 58, 59]. This clinician-rated scale contains 10 questions, each item rated from 0 (no symptoms) to 4 (extreme symptoms) [60]. The psychometric properties of the Persian version of Y-BOCS are approved in previous studies [23, 25, 59].

The total score of the Y-BOCS difference between the baseline and the week 12 among the two groups was the primary outcome measure of the trial.

We used Wisconsin Card Sorting Test (WSCT) to examine participant’s executive function at weeks 0 and 12 of therapy. The WCST was developed by Berg and Grant to assess flexibility in thinking and shifting to a new response to changing environmental contingencies [61]. It is used as a measure of executive function [62, 63]. The WCST consists of four stimulus cards and the subject receives two sets of 64 response cards. The subject should match response cards to the stimulus cards and receive feedback whether he or she is right or wrong on each trial. Important scales in the WCST include the number of categories achieved, the number of perseverative errors, and the number of set-loss errors [64].

The difference of each scale of the WCST between baseline and the end of the trial between the two groups were measured to assess the executive function of participants.

Moreover, adverse effects were monitored each four weeks using a systematic questionnaire and three open questions to include any other side effects not included in the questionnaire. In case of observation of any serious adverse effects during the course of therapy, a physician assessed the potential role of the medication in inducing the adverse effects and omitted the patient from the trial.

Missing data was imputed with last observation carried forward (LOCF) method.

### Sample size and statistical analysis

With a between-group difference of five points in Y-BOCS score, type I error of 5% and power of 90%, using G-power 3.1.9.2 we calculated a sample size of 58 (29 in each group). Considering a drop-out rate of 20%, our final sample size was calculated 70 (35 in each group).

IBM SPSS Statistic 16.5 (IBM Corporations, Somers, New York, USA) was used for the statistical analysis. Continuous variables were reported as mean±SD and categorical variables as n (%). Mean differences (MDs) between groups were reported as MDs (95% confidence interval (CI)). Fisher’s exact test, or χ2-test was used for the comparison among categorical variables. The independent samples t-test was conducted for the comparison of continuous variable values, respectively. The comparison of Y-BOCS total and subscale score changes and WCST scale scores in and between groups during the 12-week course of study was achieved by performing General linear model repeated measures. Whenever sphericity of the data could not be assumed using the Mauchly’s test of sphericity, the homogeneity of the variance is tested with Levene’s test. Score changes from baseline in the participants of each group was examined using the paired sample t-test. A p-value level of ⩽5% was defined as significant.

## Results

### Participants

**Fig. 1:**
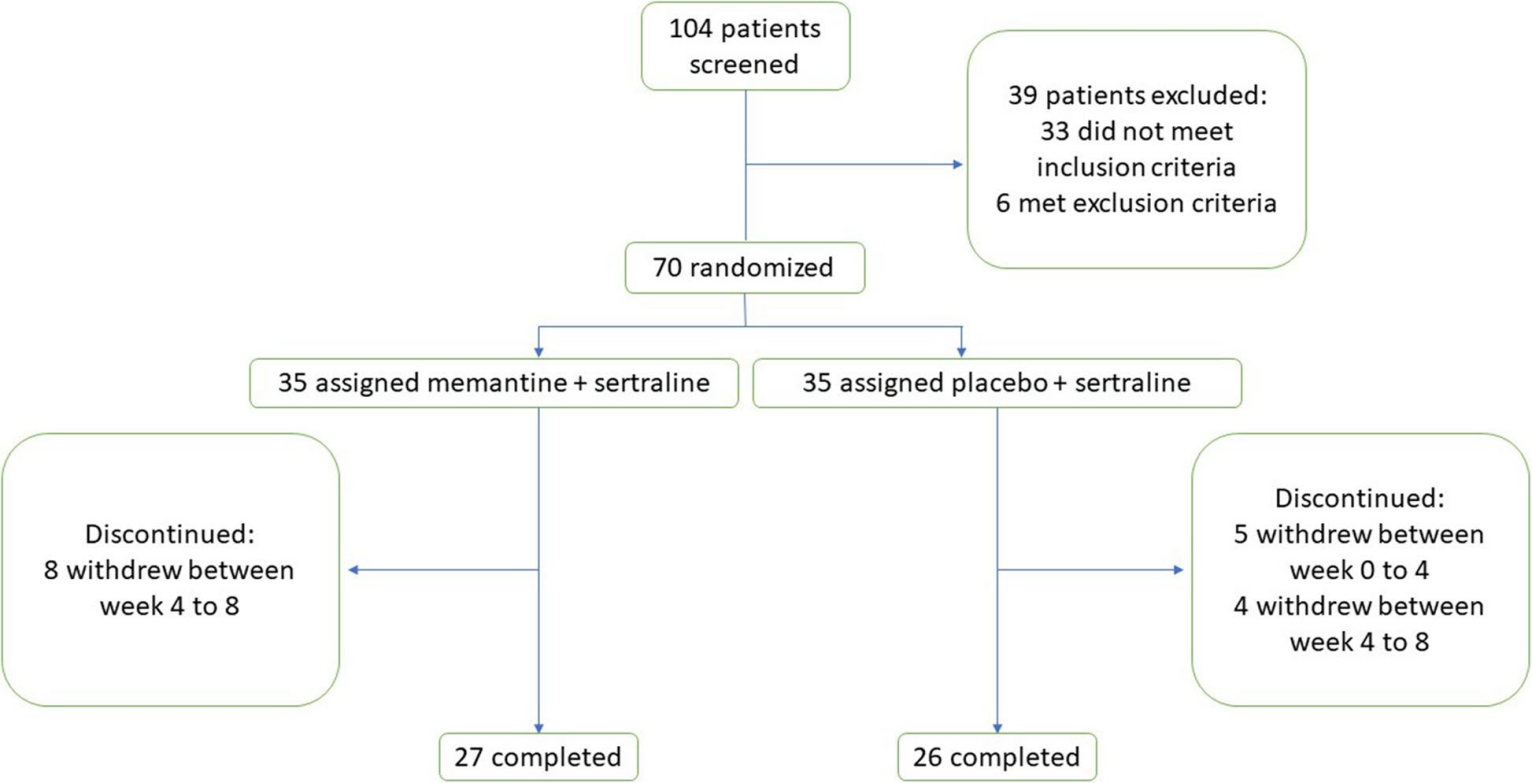
Trial participants’ flow-diagram

One hundred and four patients were screened primarily, while 70 patients were recruited (randomly assigned to groups of memantine+sertraline or placebo+sertraline), and 53 patients completed the trial. Trial flow diagram and number of dropouts are represented in Fig. 1. None of the dropouts was in regard of adverse effects or substance use. In first 4 weeks there were 35 patients in memantine group and 30 patients in placebo group that the Baseline characteristics of each group are summarized separately in Table 1.

**Table 1:** Baseline characteristics of participants in first 4 weeks

		Treatment Group				
		memantine+sertraline (*n *= 35)		placebo+sertraline (*n* = 30)		*P* value
		Mean±SD	Count (%)	Mean±SD	Count (%)	
Age (years)		35.03±11.35		334.83±10.30		
Gender	Female		27 (77.1%)		17 (56.7%)	0.07
	Male		8 (22.9%)		13 (43.3%)	
Education	Illiterate		0 (0.0%)		1 (3.3%)	0.30
	Primary		1 (2.9%)		2 (6.7%)	
	Secondary		9 (25.7%)		5 (16.7%)	
	High school diploma		9 (25.7%)		13 (43.3%)	
	University Education		16 (45.7%)		9 (30.0%)	
Marital status	Single		18 (51.4%)		11 (36.7%)	0.08
	Married		14 (40.0%)		17 (56.7%)	
	Divorced		0 (0.0%)		2 (6.7%)	
	Separated		3 (8.6%)		0 (0.0%)	
Employment	Employed		18 (51.4%)		18 (60.0%)	0.71
	Unemployed		7 (20.0%)		4 (13.3%)	
	Housewife		10 (28.6%)		8 (26.7%)	
Previous treatment	Yes		4 (11.4%)		2 (6.7%)	0.50
	No		31 (88.6%)		28 (93.3%)	
Y-BOCS score (week 0)	Total	27.88±5.65		30.11±5.79		0.12
	Obsession	15.07±2.57		15.53±2.50		0.46
	Compulsion	12.70±4.30		14.57±3.81		0.07
WCST score (week 0)	Error	22.89±9.85		22.09±10.63		0.80
	Categories	3.36±1.77		3.90±1.86		0.35
	Perseveration	6.15±5.54		7.88±9.02		0.47

*SD *standard deviation, *Y-BOCS* Yale-Brown Obsessive-Compulsive Scale

### Y-BOCS total score

**Fig. 2:**
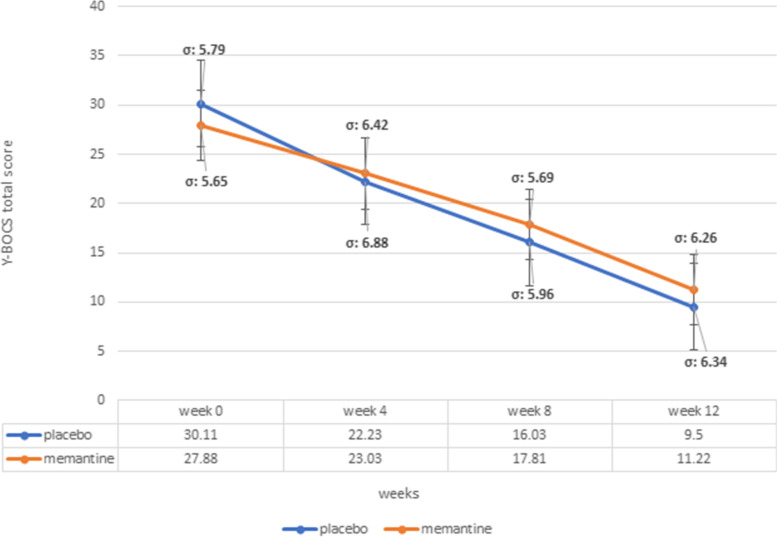
Yale-Brown Obsessive-Compulsive Scale (Y-BOCS) total score trend for each group during the trial course

The baseline Y-BOCS total score’s difference was not significant between the groups (MD (95% CI) = -2.23(−5.07–0.61), p-value=0.12, Table 1). Total Y-BOCS score changes from baseline in memantine group at fourth and 12^th^ week of the study was MD (95% CI) = 4.85 (1.77–7.92) (p-value<0.001) at week 4 and MD (95% CI) = 16.66 (13.62–19.69) (p-value<0.001) at 12^th^ week, respectively. Similarly, participants in the placebo group experienced significant Y-BOCS total score drop at fourth and 12^th^ week into the trial (MD (95% CI) = 7.88 (4.48–11.27) (p-value <0.001) in the 4^th^ week and MD (95% CI) = 20.61 (17.35–23.86) (p-value<0.001) in the end) General linear model repeated measures revealed no significant difference for the time between memantine and placebo groups (p-value= 0.71) (Figure 2, Table 2).

**Table 2:** Comparison of Yale-Brown obsessive-compulsive scale (Y-BOCS) subscales score change from baseline for treatment groups

Y-BOCS subscale score reduction		Treatment group					
		memantine+sertraline			placebo+sertraline		
		Mean±SD	MD (95% CI)	*p*-Value	Mean±SD	MD (95% CI)	*p*-Value
Total	Week 4	23.03±6.42	4.85 (1.77-7.92)	<0.001	22.23±6.88	7.88 (4.48-11.27)	<0.001
	Week 12	11.22±6.26	16.66 (13.62-19.69)	<0.001	9.5±6.34	20.61 (17.35-23.86)	<0.001
Obsession	Week 4	12.55±3.01	2.62 (1.20-4.03)	<0.001	11.73±3.67	3.80 (2.13-5.46)	<0.001
	Week 12	6.29±3.33	8.88 (7.38-10.37)	<0.001	5.3±2.76	10.23 (8.82-11.63)	<0.001
Compulsion	Week 4	10.37±4.16	2.33(0.15-4.50)	0.03	10.42±4.15	4.15 (2.01–6.28)	<0.001
	Week 12	4.90±3.42	7.80 (5.77–9.82)	<0.001	4.15±3.92	10.42 (8.34–12.49)	<0.001

### Y-BOCS obsession subscale score

**Fig. 3:**
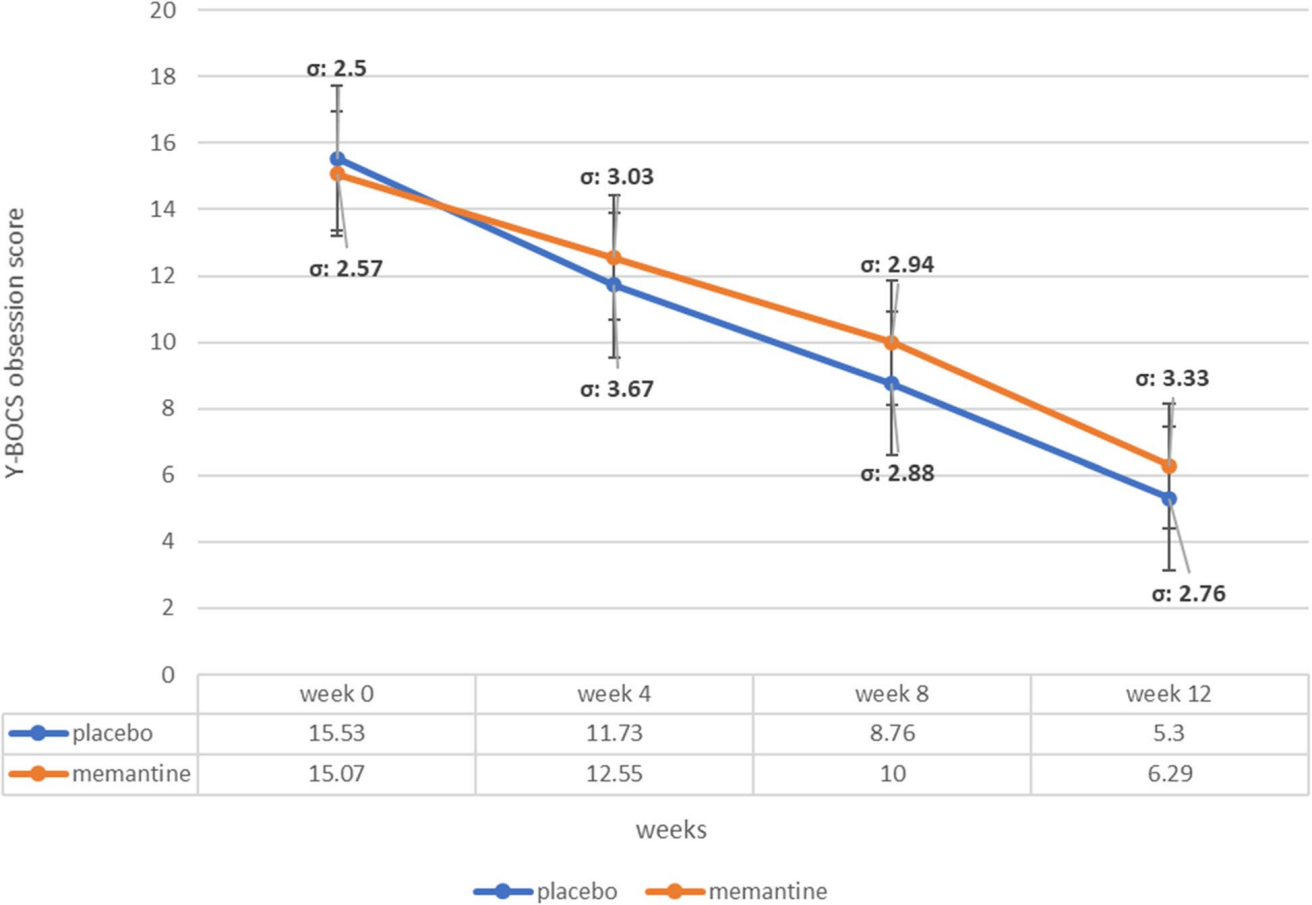
Yale-Brown Obsessive-Compulsive Scale (Y-BOCS) obsession subscale score trend for each group during the trial course

The baseline Y-BOCS obsession subscale score was not significantly different among treatment groups (MD (95% CI) =-0.46 (-1.72-0.80), p-value=0.46 (Table 1)). Obsession Y-BOCS score changes from baseline in memantine group at fourth and 12^th^ week of the study were MD (95% CI) = 2.62 (1.20–4.03) (p-value<0.001) at week 4 and MD (95% CI) = 8.88 (7.38–10.37) (p-value<0.001) at 12^th^ week, respectively. Similarly, participants in the placebo group experienced significant Y-BOCS total score drop at 12 weeks into the trial, while their score change mean differences were MD (95% CI) = 10.23 (8.82–11.63) (p-value <0.001) and in the 4^th^ week (MD (95% CI) = 3.80 (2.13–5.46) (p-value<0.001)) respectively. The time×treatment group interaction analysis by general linear model repeated-measures revealed no significant difference between groups (p-value= 0.33) (Fig. 3, Table 2).

### Y-BOCS compulsion subscale score

**Fig. 4:**
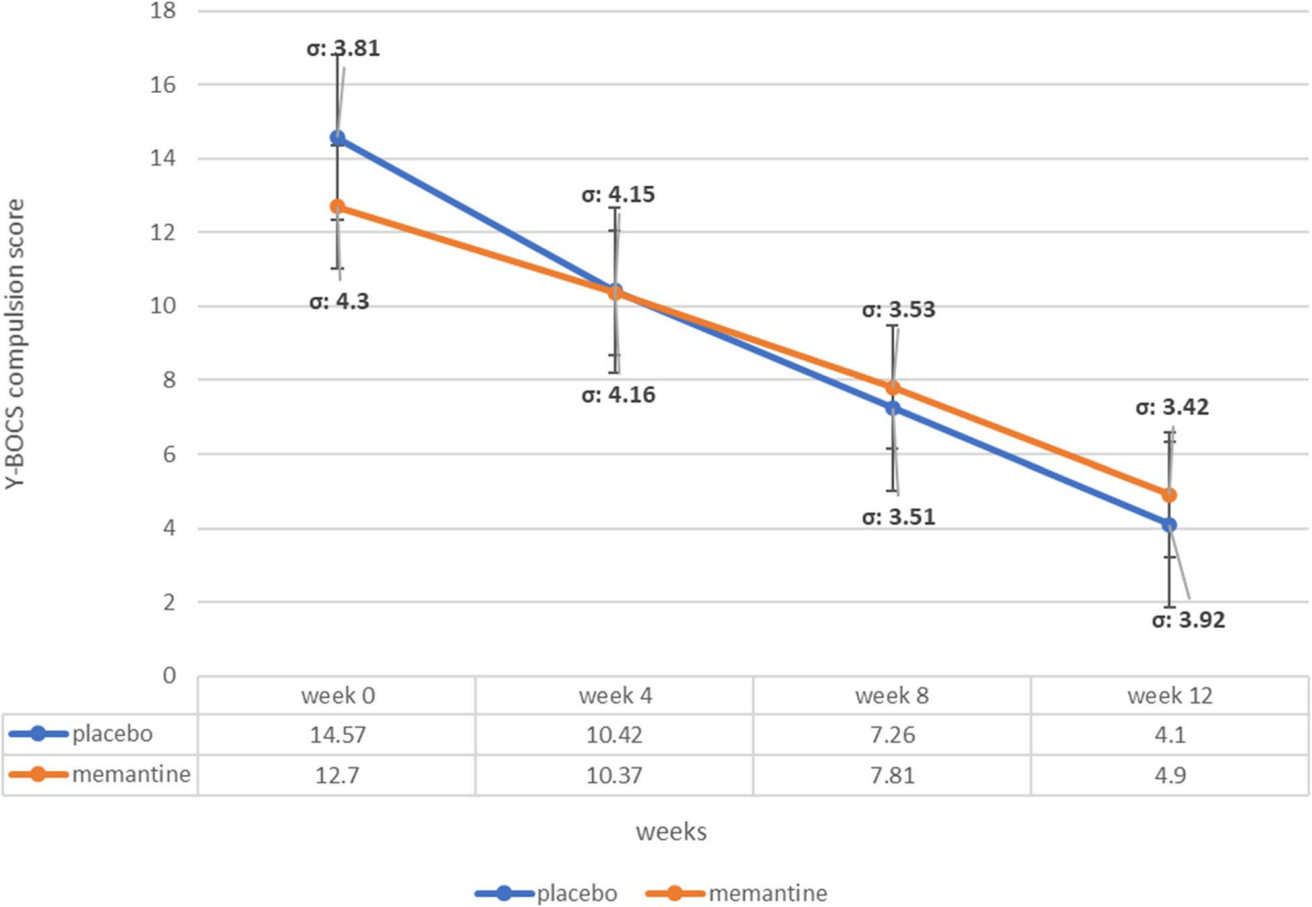
Yale-Brown Obsessive-Compulsive Scale (Y-BOCS) compulsion subscale score trend for each group during the trial course

The baseline Y-BOCS compulsion subscale score was not significantly different among treatment groups (MD (95% CI) =-1.87 (-3.89_0.15), p-value=0.07 (Table 1)). Compulsion Y-BOCS score changes from baseline in both groups at 4^th^ and 12^th^ week of the study. Memantine group difference in 4^th^ was MD (95% CI) = 2.33 (0.15–4.50) (p-value 0.03) and MD (95% CI) = 7.80 (5.77–9.82) (p value< 0.001) in the 12^th^ week and placebo group experienced significant Y-BOCS compulsion score drop at week (4 MD (95% CI) = 4.15 (2.07–6.28) (p-value<0.001)) and in week 12 (MD (95% CI) = 10.42 (8.34–12.49) (p-value<0.001)), respectively but the time×treatment group interaction analysis by general linear model repeated-measures revealed no significant difference (p-value=0.87) (Fig. 4, Table 2).

### WCST number of errors subscale score

**Fig. 5:**
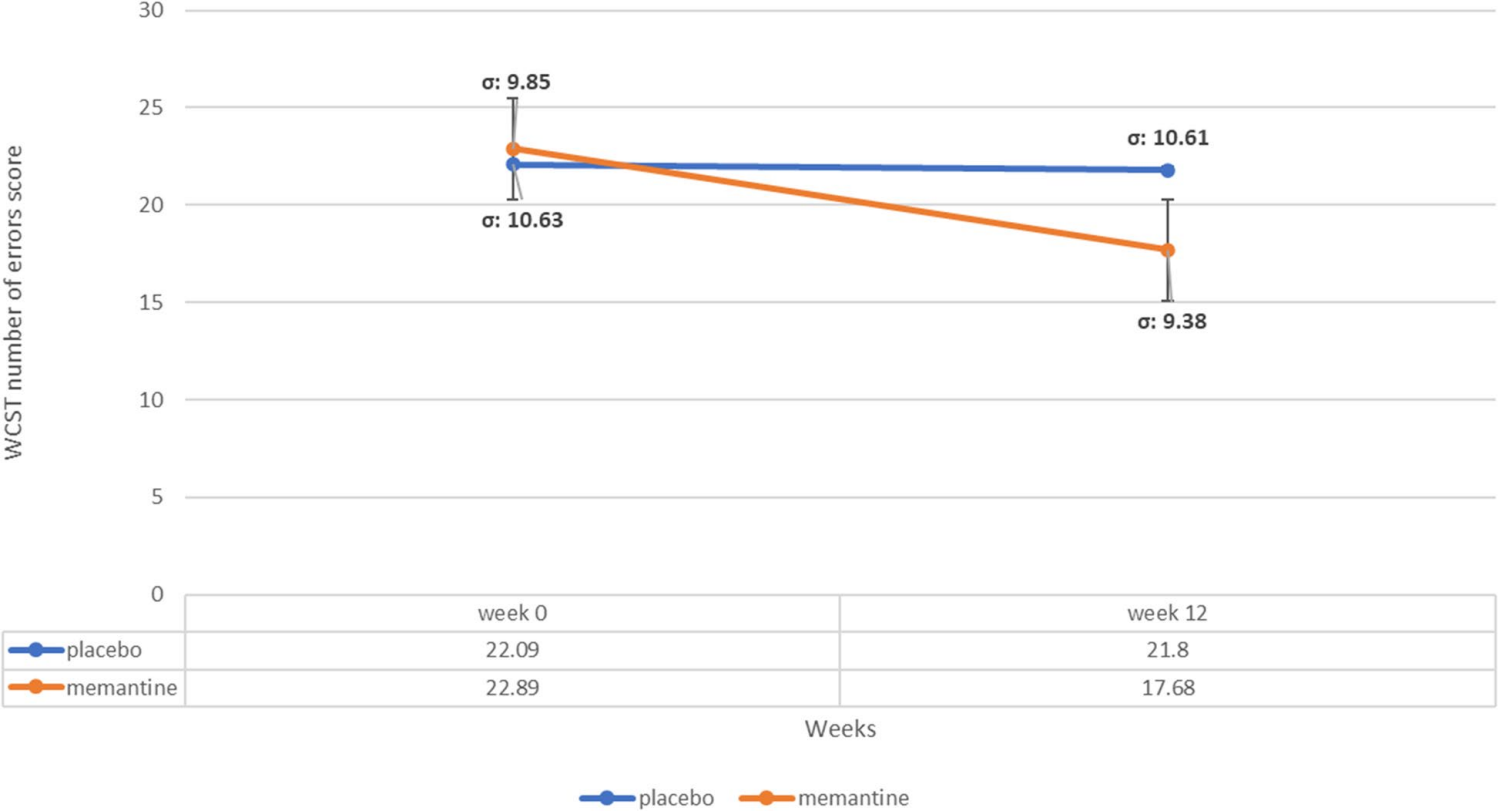
WCST number of set-loss errors subscale score trend for each group during the trial course

The baseline WCST number of errors subscale score did not significantly differ among treatment groups (MD (95% CI)=0.80 (-5.78-7.38), p-value=0.80 (Table 1)). WCST number of errors score reduced in memantine group (MD (95% CI)=5.21 (-1.11-11.53)) between week 0 to 12 but the difference was not significant (p-value=0.10). It did not change significantly in placebo group in the course of the trial. (MD (95% CI)=-0.29 (-5.73-6.32), p-value=0.92) and general linear model repeated measures revealed no significant difference between two groups (p value= 0.53) (Figure 5).

### WCST number of categories subscale score

**Fig. 6:**
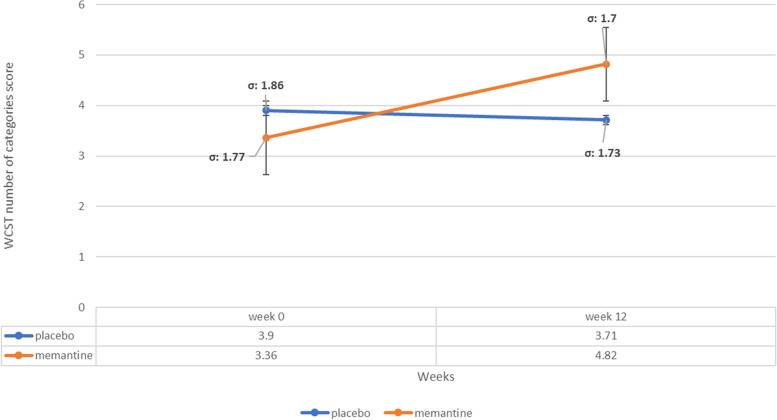
WCST number of categories subscale score trend for each group during the trial course

The baseline WCST number of categories subscale score did not significantly differ among treatment groups (MD (95% CI)=-0.54 (-1.70-0.62), p-value=0.35 (Table 1)). WCST number of categories score changed significantly between week 0 to 12, in memantine group (m_1_=3.37, SD_1_=1.77, m_2_=4.84, SD_2_=1.71, (MD (95% CI)=-1.48 (-2.62- -0.33), p-value=0.01) but it did not change significantly in placebo group (m_1_=3.9, SD_1_=1.87, m_2_=3.7, SD_2_=1.74, MD (95% CI)=0.19 (-0.93-1.31), p-value=0.73) and general linear model repeated measures revealed significant difference between two groups (10.938 (1.38)=0.77 p-value<0.001, effect size of the mean difference=0.9) [65] (Figure 6).

### WCST number of perseverative errors subscale score

**Fig. 7:**
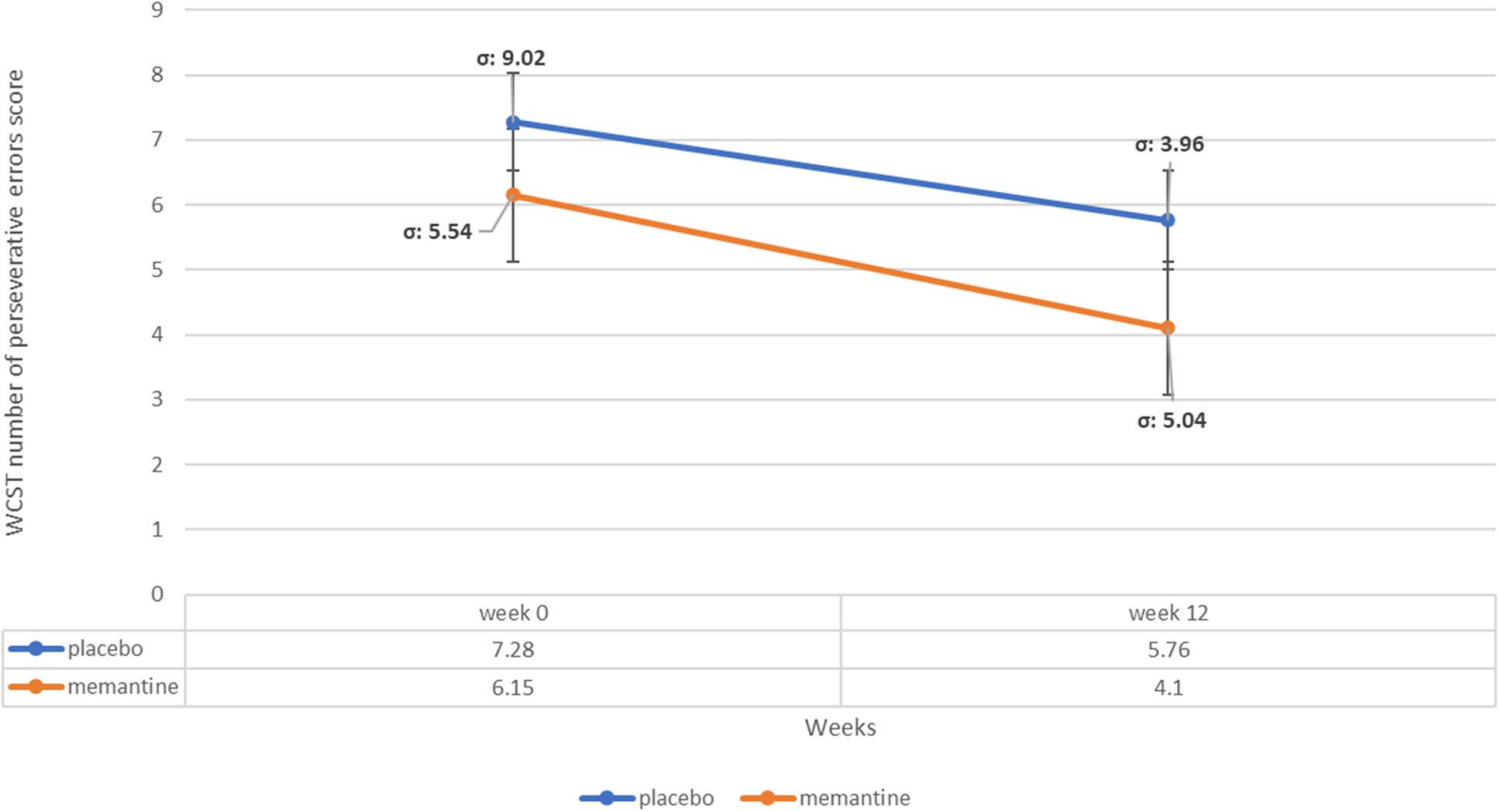
WCST number of perseverative errors subscale score trend for each group during the trial course

The baseline WCST number of perseveration subscale score did not significantly differ among treatment groups (MD (95% CI)=-1.73 (-6.58-3.12), p-value=0.47 (Table1)). WCST number of perseveration score did not change significantly between week 0 to 12, in both groups (MD (95% CI)=2.05 (-1.43-5.53), p-value=0.24 in memantine group and MD (95% CI)=2.12 (-2.22-6.48), p-value=0.33 in placebo group) and general linear model repeated measures revealed no significant difference between two groups (p value= 0.40) (Figure 7).

### Adverse effects

Adverse events were recorded during the study. Side effects were mild and did not result in withdrawal. Frequency of side effects was not different between the two groups (Table 3).

**Table 3:** Frequency of adverse events in the two groups

Adverse events	Trearment group				
	memantine+sertraline		placebo+sertraline		*p*-value
	N	%	N	%	
Muscle pain	2	5.7	2	6.7	0.87
Headache	2	5.7	2	6.7	0.87
Diarhea	1	2.9	0	0	0.35
Constipation	0	0	0	0	1
Decreased libido	5	14.3	3	10	0.60
Decreased appetite	5	14.3	3	10	0.60
Lightheadness	1	2.9	0	0	0.35
Palpitation	0	0	1	3.3	0.28
Insomnia	3	8.6	0	0	0.10
Vomiting	3	8.6	1	3.3	0.38

## Discussion

The current clinical trial don`t show a significant difference in the improvement of severity of symptoms of patients with moderate to severe OCD with augmentation of memantine (10mg/ twice per day) to sertraline through 12 weeks of the study. Although, the findings indicate a significant improvement of the patients of memantine arm in the number of completed categories of WSCT, in comparison with the placebo group. Interestingly, observed adverse effects were not suffering nor life-threatening in both groups and none of the adverse effects was, significantly, higher in the memantine group than the placebo group.

To the best of our knowledge, four previous randomized-placebo controlled clinical trial have investigated the efficacy of memantine as an adjunctive agent to standard serotonergic medications for the treatment of OCD and reported controversial findings of its efficacy, whereas this is the first 12-week double-blind, placebo-controlled clinical trial to evaluate the efficacy of memantine on the severity of symptoms as well as the cognitive function of patients with moderate to severe OCD, as an augmentation to sertraline.

Our results are in agreement with Farnia et al. report that investigated the efficacy of memantine plus fluoxetine in an 8-week, three arms trial with gabapentin plus fluoxetine and placebo plus fluoxetine in outpatients with OCD. Similar to our report, they didn`t show a significant difference between arms based on neither YBOCS total score nor response rate [53]. We report the same finding in our 12-week trial about the augmentation of memantine to another approved SSRI, sertraline. Nevertheless, Ghaleiha et al. reported memantine (10 mg/ twice per day) plus fluvoxamine more efficient than placebo plus fluvoxamine in an 8-week double-blinded, randomized, controlled trial among thirty-eight patient in the treatment of severity of symptoms and response rate of patients with moderate to severe OCD patients. In agreement with our trial, they observed no significant adverse effect in the memantine group in comparison with the placebo group [66]. Our trial provided longer follow-up as well as, to some extent, a larger sample size. The inconsistency of these two trials might be on account of augmenting memantine to different medications.

Modarresi et al. investigated the efficacy of memantine (10 mg/ twice per day) as an augmented agent for the treatment of patients with Serotonin Reuptake Inhibitors (SRIs) treatment-refractory OCD among thirty-two participants in a 12-week trial. Moreover, they indicated a significant reduction of severity of symptoms based on YBCS as well as more response rate in the memantine group than the placebo group. In addition, similar to our findings, they reported memantine as a well-tolerated and safe agent [54]. The results of their study are hardly comparable to ours due to substantial differences between recruited participants. Their trial was performed among SRIs treatment-refractory patients, while we recruited patients with moderate to severe symptoms with non-refractory OCD. Standard medications were mixed among both groups, whereas we used sertraline with the same dose among both groups to elaborate more comparable results between each group.

In the same vein, Haghighi and his colleagues performed a 12-week placebo-controlled trial on 29 inpatients with OCD to evaluate the efficacy of memantine (5-10 mg/ day) as an adjunctive agent to an SSRI or clomipramine. They reported YBOCS decreased, significantly, in the memantine group in comparison with the placebo group [58].

Two trials of Bakhla et al., and Aboujaoude et al., are not easily comparable with our study due to their different designs as open-label trials [12, 51]. Moreover, study of Stewart et al., is a single-blinded case-control study that cannot be compared with our study as double-blinded controlled trial. As previously mentioned, findings of the study is not easily comparable with our findings due to difference of setting, the dosage of memantine, as well as standard medications.

Although some of the evidence presented supports the efficacy and safety of glutamatergic medications like memantine in the treatment of OCD patients, a recent review article suggested that more well-conducted in vivo and basic experimental studies are necessary [29].

Additionally, we evaluated the effect of memantine as an augmentation to sertraline for improvement of cognitive impairment of patients with moderate to severe OCD that is one of the most disabling manifestations of this neuropsychiatric disorder [8, 9, 67]. To the best of our knowledge, our study is the first double-blind, placebo-controlled, clinical trial for this purpose. While the efficacy of the agent on cognitive impairment of other neuropsychiatric conditions, more specifically Alzheimer’s disease is well- known [68], our findings showed a probable efficacy of augmentation of memantine to sertraline for OCD patients. However, more well-designed studies with a larger sample size and longer follow-up periods are necessary.

In this study, we showed that adding memantine to the treatment reduced (although insignificantly) the number of errors and improved the number of completed categories of WCST. However, it did not change the number of perseverative errors of the subjects. According to the finding we cannot claim an improvement in executive functioning of the patients, because not all of the measures have improved. The marginal improvement in the total number of errors might be due to improvement of attention that in its turn has resulted to a betterment of learning the test mechanism and a resulting significant increase in the number of completed categories [69].

Interestingly, the number of completed categories automatically improve in normal subjects who perform WCST for a second time. This improvement seems to be related to learning the mechanism of test. In our study, this improvement only happens in the memantine group and not in the control group. Therefore, we hypothesize that a better attention and implicit learning of the test mechanism might be the underlying mechanism for the observed improvement [70]. Further research is needed to test if really attention and other cognitive measures change in the patients with OCD after using memantine.

## Limitations

Despite numerous strengths of the current study, there are some important limitations for our trial that should be considered. Although the sample size of our study was larger than previous studies, this is a clinical trial with small sample size. In addition, as we know, based on delayed response in OCD patients in comparison with some other neurotic psychiatric disorders like depression, a 12-week follow-up seems to be a short time to observe the efficacy of the treatment on the severity of symptoms and cognitive functioning of the patients. Moreover, we only used WCST to examine the executive function and cognition of our participants. Using multiple tests to examine different aspects of cognition and using functional brain imaging and electroencephalography can be helpful. Therefore, designing multi-center trials with a large sample size, with multiple tools to examine cognition and executive function, and longer follow-up is suggested. The effect of improved cognition on quality of life of patients was not the purpose of our study and was not examined. Finally, we recruited patients with non-refractory OCD in the study, and generalization of the findings to this group of patients is not reasonable.

## Conclusion

Our findings suggest a probable effect of memantine as adjuvant therapy to sertraline on executive function of patients with OCD in comparison with placebo as well as the safety and tolerability of the memantine in these patients. Nevertheless, the current results don`t support the efficacy of memantine as an adjunctive agent to sertraline for improving the severity of symptoms among patients with OCD. Based on mixed results about the efficacy of memantine on OCD symptoms, further trials are necessary.

## Data Availability

The datasets used and analyzed during the current study are available from the corresponding author on reasonable request.

## Abbreviations

OCD: Obsessive-Compulsive Disorder
NMDA: N-Methyl-D-aspartate
SSRI: selective serotonin reuptake inhibitor
DSM–5: Diagnostic and Statistical Manual of Mental Disorders
Y-BOCS: Yale-Brown obsessive compulsive scale
WCST: Wisconsin Card Sorting Test
CBT: Cognitive Behavioral Therapy
CSTC: Cortico-striato-thalamo-cortical
CSF: Cerebrospinal fluid
TL: Temporal lobe
RCT: Randomized controlled trial
MD: Mean difference
CI: confidence interval
SD: standard deviation
